# Epidemiology and Management of Atrial Fibrillation and Stroke: Review of Data from Four European Countries

**DOI:** 10.1155/2017/8593207

**Published:** 2017-05-28

**Authors:** Andreea D. Ceornodolea, Roland Bal, Johan L. Severens

**Affiliations:** ^1^eMbrace Institute, Amsterdam, Netherlands; ^2^Institute of Health Policy & Management, Erasmus University Rotterdam, Postbus 1738, 3000 DR Rotterdam, Netherlands; ^3^Institute for Medical Technology Assessment, Erasmus University Rotterdam, P.O. Box 1738, 3000 DR Rotterdam, Netherlands

## Abstract

In Europe, 1–3% of the population suffers from atrial fibrillation (AF) and has increased stroke risk. By 2060 a doubling in number of cases and great burden in managing this medical condition are expected. This paper offers an overview of data on epidemiology and management of AF and stroke in four European countries as well as the interconnection between these dimensions. A search index was developed to access multiple scientific and “grey” literatures. Information was prioritised based on strength of evidence and date. Information on country reports was double-checked with national experts. The overall prevalence of AF is consistent across countries. France has the lowest stroke incidence and mortality, followed by Netherland and UK, while Romania has higher rates. GPs or medical specialists are responsible for AF treatment; exception are the special thrombosis services in the Netherlands. Prevention measurements are only present in UK through screening programs. Although international and national guidelines are available, undertreatment is present in all countries. Despite differences in healthcare systems and management of AF, epidemiology is comparable between three of the countries. Romania is an outlier, by being limited in data accessibility. This knowledge can contribute to improved AF care in Europe.

## 1. Introduction

**Figure 1:**
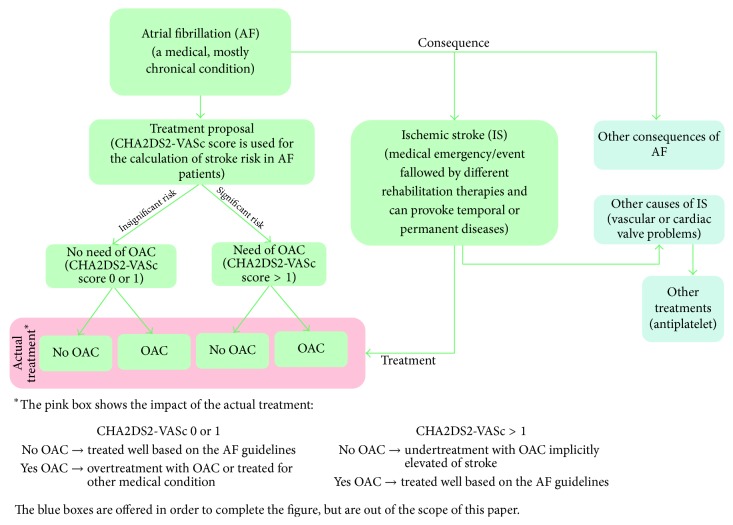
Healthcare path of AF medical condition.

Atrial Fibrillation (AF) is a common form of heart rhythm disorder (arrhythmia). In Europe, AF is detected in 1-2% of the population and is age-dependent, increasing in the elderly [1–7]. AF is mostly asymptomatic, but due to the improper flow of blood and the appearance of blood clots, it has acute risk factors such as stroke [4–6, 8].  Figure 1 presents the healthcare path of AF medical condition. Without special detection programs, stroke is, in many cases, the first sign of AF [3, 9]. The risk of a blood clot can be assessed using the CHA_2_DS_2_-VASc score and the treatment for blood clots and stroke prevention is done using oral anticoagulation (OAC) treatment [10]. Most commonly used OAC drugs in the treatment of detected AF are Vitamin K Antagonists (VKA) or New Oral Anticoagulants (NOAC) [2, 3, 9]. Overtreatment (taking OAC treatment without needing it, CHA_2_DS_2_-VASc score 0 or 1) and undertreatment (not taking OAC treatment when needing it, CHA_2_DS_2_-VASc score > 1) are frequent [3, 8, 9, 11]. Undertreatment with OAC drugs on the longer term causes severe and costly complications, such as stroke (see Figure 1). Overtreatment can also have severe and costly consequences such as bleeding. Overall, AF patients have a fivefold higher risk of developing stroke [4, 6, 12]. The most frequent stroke type in AF patients is ischemic stroke (IS), which is connected to higher death rates or worse prognosis at higher cost [4, 13].

The heaviest burden in managing and treating AF patients is the predicted doubling in number of patients suffering from AF [5, 7, 12]. In addition to the growing number of new medical cases and increased risk of stroke in AF patients, there is another challenge, namely, the large discrepancy between and within countries in management and outcome of AF patients [8, 9, 12, 14, 15]. These differences in management of the medical condition, despite the existence of communal guidelines for AF management [1, 2, 16, 17], reveal unequal access to AF treatment for patients with the same health conditions within Europe [8]. All these challenges on treating and managing the AF patients have major public implications and high increase in costs in the long-term [7, 8].

In order to reduce the burden of AF management and prevent stroke at international level, it is essential to have actual knowledge of the AF data in the different countries [15]. This paper aims to bring forward this type of information by offering a collection of data on epidemiology and management of AF and stroke in several European countries.

Although numerous studies offer an overview on epidemiology of AF and stroke in different countries [14, 18–20] or on the management of AF medical condition [2, 3, 12, 14, 21, 22], this paper brings a unique perspective. It brings an original and complete review of available literature on both epidemiology and management of AF and stroke in different countries and indicates the interconnection between the two dimensions. The scope of this paper is (1) to undertake a narrative review of literature on available data and (2) to offer a descriptive analysis (on magnitude) of the most recent (by recent we mean information from our literature study with the time window 2005–2016) and trustworthy (by trustworthy we mean official healthcare organizations and international journal publications) available data on the epidemiology and management of AF and stroke in four different European countries.

*Countries in Focus.* The focus of this paper is on four particular European countries: United Kingdom, France, Netherlands, and Romania. The choice of the countries has been made in order to consider (1) different type of countries (as geographic placement and economic level) as well as (2) different type of health care systems and organization of the medical care.

These countries can also be seen as prototypes for the other countries in their region, with the UK for west European countries and RO for the East European ones and NL for the north and FR for the south European countries. In addition, the organization of the health care system in each of these countries was considered. In what follows, we present the type of healthcare system (tax based versus insurance based; regionalized/decentralised versus centralized healthcare system) and role of the GP in the organization of care (functions as a gatekeeper, yes or no) for each country in focus.

In the UK, the public healthcare system provides free of charge healthcare, with easy access for all permanent residents to primary care (general practitioner). Although the National Health Service (NHS) covers four different countries (England, Scotland, Wales, and Northern Ireland), it is a universal system that emphasises on Predictive, Preventive, and Personalized Medicine elements [23]. This system is the responsibility of the government and it is largely tax funded; it is a patient-centred system based on need instead of ability to pay. In UK, GPs function as gatekeepers and they regulate and coordinate primary and secondary medical care [23].

In contrast, the French healthcare system is based on the Bismarckian model with influence from the Beveridge model [24]. The main characteristics of the French decentralised healthcare system are the high level of population health, the degree of freedom for physicians and patients, the absence of waiting lists for treatment, and the universal coverage [25]. The accessibility to the health care in France makes the role of the GP as gatekeeper not extremely strong; population has the opportunity to access specialized care directly for a higher cost than with prescription from the GP [26].

Regarding the Netherlands, this small country has a unique and innovative centralized healthcare system as a consequence of the 2006 healthcare reform. This system is based on a single compulsory healthcare insurance scheme. For both providers and insurances, managed competition is the main driver of the healthcare system; GPs function as gatekeepers to the system and patients are in principle free to choose their insurer and providers of care [27].

Finally, Romania is a large East European country with a former communist background; it has a lower economic level than the countries presented above. The previous Semashko health system has yet to be fully developed in the long period of democratic transition. In the process of decentralisation of the system, numerous healthcare reforms took place due to the political, social, and economic changes. The health system is tax based and GPs function as gatekeeper. However, Romania has an overall poor health status with a life expectancy lower and infant mortality higher than the average European rate [28].

## 2. Methods

This paper offers a narrative review of the literature on AF and stroke. The search of literature has been done per country, using a specially developed topic index. The order of the countries in the research is as follows: France, UK, the Netherlands, and Romania. The search of data combined different sources of information: (1) scientific literature, (2) “grey” literature including governmental reports and national/regional working documents, and (3) websites and databases of different (inter)national healthcare organizations.  Table 1 names the literature sources used per country.

**Figure 2:**
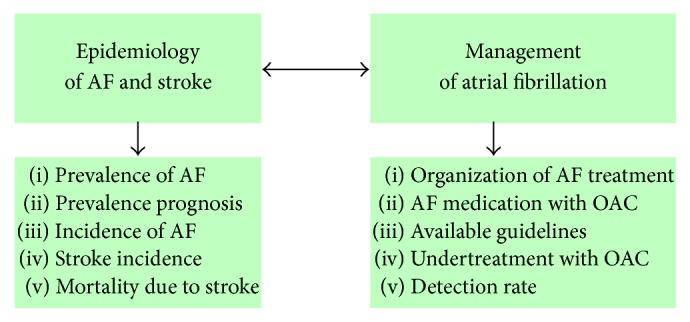
Interconnection dimensions in focus.

A specially designed topic index was used by first author in order to extract data per country. The search index includes terms on epidemiology of AF and stroke, and management of AF with OAC treatment (see Figure 2). Full disclosure of the search strategy and examples of the keywords used in the search are presented in Appendix. The epidemiological data considered are prevalence and prevalence prognosis of AF, incidence of AF, stroke incidence, and mortality due to stroke. Although AF is mainly associated with IS, we focused on general stroke data, as no heterogeneity between studies in IS data was found. The domains in the management of AF considered are organization of treatment, medication, available guidelines, level of undertreatment, and detection rate.

The index terms were researched in English language per country, for the time window 2005–2016, using different search engines: PubMed, SAGE, BMG, ScienceDirect, Global Health Data Exchange, Oxford University Press Journals/Europace, Research Gate, and Google (Scholar). As such, specialized international AF Journals such as American Hearth Journal, European Hear Journal, Journal of Cardiology, Stroke, Heart Journal, and Chest Journal were included in the search. Besides, the “grey” literature was consulted, including governmental reports and national/regional working documents for each of the countries (in their domestic language). In addition to the literature presented above, websites and databases of the different national and international healthcare organizations (in their domestic language) were used in order to supplement the missing data. Where country specific data was not found available, the research was enlarged to regional studies or clustered country studies for data enrichment. This strategy was especially used for the epidemiology and management of AF in Romania where published information is limited. Full disclosure of the search strategy is available in Appendix.

In order to be used, all relevant research findings have been prioritised based on two criteria: (1) the most recent data and (2) the strength of evidence (trustworthiness of sources). These two criteria functioned also as criteria for exclusion of some data over the other. The time interval set for this literature review is from 2005 till beginning of 2016, with recent data (cross-checked between articles) having priority. The most trustworthy sources of information considered were official healthcare organizations and international journal publications.

After collecting the data per country, these were checked and enhanced with comparative literature from studies on multiple countries/regions. For example, due to limited information available on Romania, the specific data was verified and enriched with information from studies on East European countries.

Around 300 articles met the initial inclusion criteria for all the four countries. From these around 100 articles were left after the first exclusion (based on relevance of title, abstract) and 50 after the second exclusion (based on full text and check of duplicate). After full reading of text by first author, a selection was done (based on usability of the data and study settings design) and in the end 30 articles were used as reference for the four countries in focus (see Table 1).

In the last phase of the research, the country specific reports have been double-checked for consensus with regard to the data authenticity and validity with professional experts from each country (see acknowledgments). Professionals were asked to assess whether the data in the country report represented the most actual and most correct data. These national experts have been chosen based on their background in AF and stroke awareness at national level, and/or their collaboration with the eMbrace Institute foundation. Unfortunately, no double-check of data was possible for Romania; although different persons and organizations were accessed, no national professional could be approached in this respect.

## 3. Results

The results of this literature study offer a collection of data on epidemiology and management of AF medical condition in patients who take or do not take OAC treatment in order to prevent stroke in four European Countries (UK, F, NL, and RO). This paper considers any type of OAC treatment available in the countries in focus (either VKA or NOAC). Tables 2 and 3 present condensed data of the four countries per domains of consideration, on epidemiology and management, respectively.

### 3.1. Epidemiology

This section includes the epidemiological data of AF medical condition and stroke, namely, prevalence and prevalence prognosis of AF, incidence of AF, stroke incidence, and mortality due to stroke.

The prevalence and prevalence prognosis of AF are meaningful values since they report the number of cases as a fraction of the whole population. The incidence of AF and the incidence of stroke report the rate of new diagnoses cases as a fraction of the population at risk.

In what follows each of the epidemiological aspects of AF and stroke will be presented one by one.

#### 3.1.1. Prevalence and Prevalence Prognosis

Prevalence of AF is reported in the literature in many specific study settings or patient groups. Although prevalence differs per age group and gender, the overall AF prevalence in the UK, France, and the Netherlands is consistent at ~1,5% [7, 29, 30] with the lowest values in NL [7]. By the year 2050, in the European Union a doubling of the AF cases is expected, especially due to the aging of the population [7, 30].

#### 3.1.2. Incidence

Incidence of AF also varies with age group. For example, in NL are reported 1.1 per 1000 person years in age group 55–59 and 18.2 per 1000 person years in age group 80–84 [13]. In UK the incidence of AF is estimated to be between 1.7 and 3 per 1000 person years [31], while in FR a little lower, at approximately 1.1 to 2.3 per 1000 person years [32]. From our knowledge, no data is available on the incidence and prevalence of AF in RO. Variation of reported AF incidence should be read with some caution as the difference between countries can largely be explained by the difference in study reported groups.

#### 3.1.3. Stroke Risk and Mortality

AF in general causes a fivefold rise in stroke risk [6, 12]. AF is mostly associated with IS which brings more complications and poorer prognosis than other types of stroke [13, 32]. Around 25% of the causes of all stroke are connected to AF [6]. As no heterogeneity between studies in IS data was found, we looked into general stroke data. The WHO estimates on stroke incidence are the lowest in FR (number 1 in Europe), followed by NL and UK [20]. Eastern European Countries (Romania included) have the highest risk of stroke in Europe [22].

Mortality due to stroke follows the same sequence as stroke incidence, with the lowest rate in FR (31 per 100,000 persons) followed by NL (35 per 100,000 persons) and UK (42 per 100,000 persons) [33]. The East European countries in general (nonspecific for Romania) have higher stroke mortality rates. These mortality rates in Eastern Europe are increasing in number lately, while in the Western European countries there is a decline in stroke mortality [20, 33].

### 3.2. Management

In the management of AF medical condition for the countries in focus we considered five aspects: organization of treatment; medication used in the treatment of AF (either VKA or NOAC); available guidelines used in the management of the disease; level of undertreatment; and detection rate programs for AF and stroke prevention. In what follows each of the management aspects will be presented one by one.

#### 3.2.1. Organization of Treatment

Organization of AF treatment is different in each of the countries, resulting in differential treatment for patients with the same health care condition within Europe [22]. The situation of the four countries in focus is presented hereunder.

In UK, the national structured healthcare system, NHS, includes implicitly the management of AF patients with OAC treatment. The GP has a 100% implication in AF patient treatment, with no special anticoagulation clinics for the management of AF medical condition. With more than 10% of the patients over 65 years old suffering from AF, GPs have an intensive task to deal with the identification as well as the management of AF on a regular bases [34]. The NICE guideline on AF builds explicitly on the relationship between GPs and their patients for a clear management plan which takes into account the patient's personal preferences and the clinician's view of evidence [35].

France has decentralised organization and management of OAC treatment. In France, patients suffering from AF are either seen by general practitioners or general cardiologists [36]. The result of three pooled cross-sectional studies in France shows that the number of GPs giving care to AF patients is almost twice as high as the number of cardiologists (5,553 GPs and 3,189 cardiologists) [37]. There are no specialized anticoagulation clinics in France, but access to laboratories and doctors is quite easy [37].

Romania also has a decentralised organization of treatment; no special clinics are available. In general, patients with AF are detected by GPs and directed for treatment and follow-up to the medical specialist, generalist, or cardiologist responsible for managing and prescribing OAC treatment [38].

In contrast to these three countries, the decentralised organization and execution of OAC treatment in NL is done through specialized outpatient clinics named “trombosediensten” (thrombolysis services). These clinics (53 in total) are responsible for the management and dosing of OAC treatment (VKA treatment) in AF patients [39, 40]. Initially, patients are diagnosed by GP or cardiologist based on clinical examination, patient history, and ECG [41].

#### 3.2.2. Medication

Regarding AF medication, no national studies on the use of one OAC treatment over the other (VKA or NOAC) are available in the countries in focus. However, regional studies show that the predominant treatment of AF patients is the traditional vitamin K treatment, the VKA. Nevertheless, it should be considered that wide geographical differences could exist. A recent study from the UK shows that 75% of the patients are treated with VKA while in France 86% [42]. NOAC is the new alternative to VKA and entered the market of all countries in 2012. This latest medication experiences a rapid increase in number of uses especially in the younger patients [2].

#### 3.2.3. Available Guidelines

Guidelines of AF management have an important role in providing medical practitioners with regulations for the best treatment of AF patients, considering the outcome and risk-benefit ratio. Also, guidelines provide equal treatment possibilities for patients with similar medical conditions across Europe. The “Guidelines for the management of atrial fibrillation” from the Task Force for Management of Atrial Fibrillation of the European Society of Cardiology (ESC) 2010/2006 [1, 16] are communal guidelines provided by the European Society of Cardiology which are available in all of the countries in focus. Also the new “2016 European Guideline on cardiovascular disease prevention in clinical practice” is expected to be adopted in all of the countries [17].

Next to this international guideline, there are also national guidelines available in all of the countries. In France, there are the “Guide Pratique d'Élaboration: Protocoles pluriprofessionnels des soins de premier recours Exemple gestion quotidienne des AVK” (PPSPR) Nov. 2011 and “Guide Parcours Arcours de Soins: Fibrillation Atriale” (HAS) from February 2014; in the UK there are the NICE clinical guidelines from 2006 and 2014; in the Netherlands there is a guideline available for the GP, from the “Nederlands Huisartsen Genootschap” (NHG), and there is a guideline from the “Kwaliteitsinstituut voor de Gezondheidszorg” (CBO). Cardiologists in NL use the ESC guideline and a specific “leidraad” (manual) for the NOAC introduction.

#### 3.2.4. Undertreatment

Undertreatment, as explained earlier, considers the cases of AF patients in need of OAC treatment (CHA_2_DS_2_-VASc score > 1) that do not benefit from the medication, having great medical implications and raising the risk of stroke [3, 11]. Although undertreatment with OAC drugs differs per study/age group, this phenomenon is present in all countries [3, 9, 11, 31]. The lowest level of undertreatment is reported in the Netherlands (18%) particularly in elderly [41, 43], and the highest is in East and South Europe (Romania included) [9, 44]. Recent studies in UK and FR also show high degrees of undertreatment [31, 37]; around half of the detected stroke patients with AF eligible for OAC treatment do not receive it, although there is proven efficiency of OAC treatment in stroke prevention [45–47].

#### 3.2.5. Detection Rate

At the same time, between a third and a half of patients affected by AF are not detected in time but are discovered in a complicated or fatal phases [47]. Several strategies of AF detection and diagnosis are present in UK at national level, where GPs function as gatekeepers. Through these programs, screening of irregular pulse check is followed by recommended electrocardiography (ECG) on all suspected AF patients (symptomatic or not) [48]. In UK, patients aged >65 are all screened based on pulse taking (eventually followed by ECG), as a systematic screening programme [49]. In FR, NL, and RO no special programs are known for the detection of AF patients in order to reduce stroke risk. Case finding in these countries is done randomly, when patients present themselves to the medical doctor with symptoms [13, 37].

## 4. Discussion

Knowledge of data in the different countries is desirable since it can contribute to reduced burden of AF management and improved medical condition of AF patients through stroke prevention in different countries. This is the first paper to undertake a review of available data on epidemiology and management of AF and stroke in these four European countries (UK, FR, NL, and RO). Learning from one another at national level and preventing stroke by fighting against undertreatment with OAC drugs in AF patients can diminish the expected burden of AF management.

### 4.1. Epidemiology

This paper shows similarities in epidemiological data between some of the countries in focus (UK, F, and NL). One resemblance in data is the prevalence of AF medical condition, which is affecting ~1,5% of the total population. These results are consistent with the findings from other European studies such as Camm et al. 2010 [1] but new compared to the world-wide study of Lip et al. 2010 [18] where high variation in AF prevalence is found.

The growth in prevalence of AF is another topic of resemblance. By the year 2050, the number of people suffering from AF is expected to increase by three times in USA [50] and by two times in Europe [7, 18, 29, 51]. A study by Krijthe et al. reports a lower prognosis of AF prevalence in the Netherlands compared with EU in general. The number of individuals with AF will more than double, to a peak of about 553,700 in the year 2050, and then decrease slightly to 547,700 in the year 2060 reflecting 3.2% of the Dutch population [7]. An explanation to this alarming prevalence prognosis estimate is connected to the aging of the population over the age of 55 [7]. This aspect is of real importance since with the growing number of cases the burden of managing the AF medical condition becomes even higher.

The highest difference in epidemiological data between countries is met in reported AF incidence. The highest values are registered in NL, with an overall rate of 9.9/1000 person years in a population over 55 years. The encountered variation in values is considered to originate from different sources of case reports considered (hospital based survey, community based studies, clinical trials, etc.), as well as from the different age groups in the study population. However, the use of the different incidence values should be used with caution.

As a general trend, patients suffering from AF face increased risk of stroke and more severe and costly complications [13]. In UK, FR, and NL the stroke incidence estimates are between the lowest 10 in Europe [20]. It is hypothesized that the lowest level of stroke, which is reported in France, is connected to diet and lifestyle [19]. Although the incidence of stroke is also reflected in the level of mortality due to stroke, we believe that these cases could be prevented or diminished through better AF case identification and in time treatment with OAC drugs.

Romania is an outlier by being limited in epidemiological data and access to medical information. The lack of data and incomplete medical records encountered in Romania have different reasons. On the one hand, there is a lack of understanding of the potential benefits of data registration of AF patients and stroke cases [15]. On the other hand, there is an overall shortage of qualified professionals and lack of incentive in data registration, although the infrastructure and the modern public health information resources are available [52].

### 4.2. Management

In the management of AF medical condition, there are similarities to a certain degree despite the different organizations of treatment and AF detection programs. The similarities are in medication, guidelines, and presence of undertreatment with OAC drugs.

In three of the countries the GP or medical specialist/cardiologist is responsible for diagnosis, treatment, and monitoring of AF patients. Unlike the other countries, the Netherlands has special local anticoagulation clinics for AF patients. These regional outpatient clinics are responsible for the management of care and dosing of AF treatment. The Dutch example of management of AF patients could be related to a lower level of undertreatment and improved therapies in stroke prevention in AF patients [18].

The reported presence of undertreatment with OAC drugs in AF patients in the different countries [3, 11, 43] has multiple and costly complications, especially in the ones with elevated risk of stroke [3, 8]. In addition, another special group known to be more prone to undertreatment with OAC drugs are the elderly [21]. In literature, the level of undertreatment is associated with nonadherence to guidelines [9, 53]. Interestingly enough, the ESC 2006 or 2010 guideline is available in all the countries in focus. Next to the international guidelines, there are also national guidelines available in most countries. However, the level of undertreatment is still significant in all the countries [3, 11, 31], with the lowest reported values in the Netherlands [41, 43]. This result could be explained by the different model of organization of treatment for AF patients in this county, and by the usability of the available guidelines [53].

Another issue that plays an important role in stroke prevention is the detection of AF patients at a prior stage. Since AF is a silent disease, mostly asymptomatic, detection programs are important to be done systematically at population level. From the countries in focus, only UK stands out with a special screening program for AF detection, implemented at national level. Intriguingly, better detection of AF cases in UK is not reflected in lower levels of undertreatment, or a reduced number of strokes and less mortality in comparison to the other countries. This issue is considered to be a topic for further study.

Romania remains an outlier in access to information, also on management and treatment strategy. The data on Romania has been enhanced with information from studies on East European countries (Romania included).

### 4.3. Strengths and Limitations

An advantage of this literature study is that it goes beyond the standard review methods by using multiple sources of information such as scientific literature, “grey” literature, and national and regional documents; the information was searched in English as well as the other national languages. The data was prioritised based on date and trustworthiness of the sources. In order to assess the authenticity and validity of the data, the reports of each of the countries have been double-checked with national experts. Unfortunately, although numerous attempts were initiated to access a national expert in Romania, no professional was available to double-check the country report. This attitude could be the effect of crowded timetables for the professionals or lack of actual knowledge of the severity of AF problem in Romania.

A drawback of this study is that the data used comes from different sources and studies. This makes a comparison between the four countries challenging, since there are multiple study settings, designs, sample sizes, and different time intervals.

Furthermore, attention should be given to the fact that although the healthcare problem presented in this paper is the same for all the different countries, the national registrations and the awareness of acting on the AF condition differ per country. This could have consequences for the actual registration and availability of the data.

Since this paper is based on a narrative literature review and offers a descriptive analysis (on magnitude) of available data on epidemiology and management of AF, no statistical analysis or meta-analysis were planned or executed. As such, this paper provides the reader with an up-to-date collection of information on epidemiology and management of AF and stroke in four European countries (NL, UK, FR, and RO). These four countries could represent prototypes for the AF epidemiology and management of the other European countries. In this way, the results are illustrative for a broader range of countries and the information provided in this study could be used in order to lower the expected burden of AF management and improve medical condition of AF patients through stroke prevention in the different countries.

## 5. Conclusion

This paper brings knowledge on the epidemiology and management of AF in four European countries: the UK, France, the Netherlands, and Romania. The healthcare system and management of AF is different between countries. However, these differences are not reflected in the reported epidemiological data of three of the four countries (UK, F, and NL). Romania is an outlier by being limited in the availability and accessibility of data. Whether the same trends and principles also apply to other East European countries would be a subject for further study.

These differences in management of the medical condition prove unequal access to AF treatment for patients with the same health conditions within Europe, despite the existence of communal ESC guidelines. The growing number of AF patients combined with the discrepancies in AF management and access to AF treatment lead to a heavy burden for the medical system and increased cost in the long-term.

*Recommendations.* Knowledge of data in the different countries can contribute to a reduced burden of AF management and improved medical condition of AF patients through stroke prevention. Using the example of the Netherlands in the organization of treatment through specialized anticoagulation clinics could result in a lower level of undertreatment and improved programs of stroke prevention. Also, the UK national program of AF detection is a desirable strategy of stroke prevention to be used in the other countries.

Practical recommendations based on this paper would be standardization of the systems with regard to organization of the AF treatment, as well as matching measurements and registries, which prove to be so necessary. In this regard, the presence of a communal clinical guidelines, as the ESC guideline, is a step forward, but action for a practical implementation and use of guidelines is also needed. All in all, learning from one country to the other could offer benefits.

## Figures and Tables

**Table 1:** Literature sources per country.

	UK	FR	NL	RO
Scientific literature study	Hobbs 2005, Krijthe 2013, Barra 2015, OECD 2011, Truelsen 2006, Le Heuzey et al. 2014, Shantsila 2015, Wilde 2006, Fitzmaurice 2005, Zhang 2012, ESC 2010	Charlemagne 2011, Cotte 2014, OECD 2011, Truelsen 2006, Touze 2005, Le Heuzey et al. 2014, Zhang 2012, Lévy 1999 ESC 2010	Krijthe 2013, Heemstra et al. 2011, OECD 2011, Truelsen 2006, Willemsen 2011, Camm 2010, Rosendaal 1996, Lip 2015, Malmivaara 2015 Arts 2013, ESC 2010	WHO, Zdrenghea 2009, Malmivaara 2015 Lip 2015 Purcarea 2009 ESC 2006, 2010

Grey literature (governmental reports and working documents)	NICE guideline 2006, 2014	PPSPR 2011, HAS 2014	NHG, CBO	—

Websites and databases of different (inter) national healthcare organizations	OECD 2011, NICE	OECD 2011	OECD 2011, Federatie van Nederlandse Trombosediensten	OECD 2011, OECD 2014

External expert	Gregory Lip	Jean-François Schved	Ron van ‘t Land	—

**Table 2:** Epidemiology considerations.

Epidemiology considerations	UK	France	Netherlands	Romania
Prevalence of AF	1,5%^*∗*^ (2004) (Hobbs 2005)	1,5%^*∗*^ (in 2005) (Charlemagne 2011)	1,4%^*∗*^ (in 2005) (Krijthe 2013)	NA

Prevalence prognosis 2050	Between 1.2 and 1.5 million (Krijthe 2013)	Between 1.1 and 2 million (Charlemagne 2011)	~554.000 (Krijthe 2013)	NA

Incidence of AF	Between 1.7 and 3 per 1000 person years (Barra 2015)	Between 1.1 and 2,3 per 1000 person years (Cotte 2014)	Between 1.1 and 18.2 per 1000 person years (Heemstra et al. 2011)	NA

Stroke incidence estimates	9004 per 100,000 (Truelsen 2006)	5999 per 100,000 (Truelsen 2006)	8530 per 100,000 (Truelsen 2006)	NA

Stroke mortality	42 per 100,000 population (2009) (OECD 2011)	31 per 100,000 population (2009) (OECD 2011)	35 per 100,000 population (2009) (OECD 2011)	In East European countries higher than in Western countries (WHO)

Each of the items per row represents the value for the country followed by the year and the reference between brackets. ^*∗*^Percentage of the whole population.

**Table 3:** Management considerations.

Management considerations	UK	France	Netherlands	Romania
Organisation of treatment	100% GP following the national guidelines (NICE 2014)	2/3 GP, 1/3 cardiologist (Touze 2005) access to biology laboratories is quite easy (Le Heuzey et al. 2014)	GP/cardiologist set the diagnose (Willemsen 2011) (Camm 2010), anticoagulation clinics responsible for monitoring and dosing (Rosendaal 1996)	GP, medical specialist, cardiologist (Purcarea 2009)

Medication	VKA used in 75% of the cases of treatment with OAC (Le Heuzey et al. 2014)	VKA used in 86% of the cases of treatment with OAC (Le Heuzey et al. 2014)	NA	NOAC or VKA 28,5% Aspirin 46% (Zdrenghea 2009)

Available guidelines	ESC 2010, NICE clinical guideline 2006, 2014	ESC 2010, PPSPR 2011, HAS Guide Parcours de Soins-Fibrillation atriale 2014	ESC 2010, NHG, CBO	ESC 2006 (2010)

Undertreatment	34% of AF patients with CHADsVASc >2 do not receive OAC treatment (Barra 2015; Shantsila 2015; De Wilde 2006)	More than 50% of stroke patients with AF do not receive OAC treatment (Touzé 2005, Kirchhof 2012)	Undertreatment with OAC drugs in the elderly (Willemsen 2011, Arts 2013)	Almost all categories of drugs are underused (Zdrenghea 2009; Lip 2015)

Detection rate	Screening programs for +65 through the GP (Fitzmaurice 2005)	Not much done (Touzé 2005)	Done aleatory, when patients present themselves with symptoms to the GP (Heemstra 2011)	Preventive measurements still lacking

Each of the items per row represents the value for the country followed by the reference between brackets.

## Appendix

### Literature Search Strategies

The search of literature has been done per country and topic index. The order of the countries in the research is as follows: France, the UK, the Netherlands, and Romania. The study of France considered the largest search index: (1) epidemiology of atrial fibrillation (AF) and stroke, (2) management of AF and use of oral anticoagulation drugs (OAC) as well as stroke prevention strategies, and (3) costs associated with AF and OAC treatment. Due to difficulties in accessing and processing data on costs within and between countries, this section has been taken out of the current study.

The search index on epidemiology included* prevalence AF*,* prevalence prognosis*,* incidence of AF*,* incidence ischemic stroke (IS)*,* incidence of IS due to AF*,* incidence of IS in AF patients who take OAC treatment,* and* mortality due to stroke*. The management section considered the following topics:* organization of treatment, medication, guidelines used for management of AF*,* guidelines used for OAC treatment and IS prevention, undertreatment, and detection rate*.

Although AF is mainly associated with IS, this study could not include this data due to the lack of heterogeneity in the available studies. However, the incidence of stroke in general and mortality due to stroke were taken into consideration. Other data not included in the final paper due to heterogeneity problems are* incidence of IS due to AF* and* incidence of IS in AF patients who take OAC treatment*.

Several topics were compressed due to similitudes under an umbrella topic. For example* guidelines used for management of AF* and* guidelines used for OAC treatment and IS prevention* were grouped in* available guidelines*.

The search strategy used in PubMed, SAGE, BMG, ScienceDirect, Global Health Data Exchange, Oxford University Press Journals/Europace, Research Gate, and Google (Scholar) included the country in focus and each of the keywords from the search index presented above, for example, 〈FranceANDepidemiologyANDatrial fibrillation〉, then 〈FranceAND“incidence atrial fibrillation”〉, then 〈FranceAND“incidence ischaemic stroke” OR “incidence ischemic stroke”〉, and then 〈FranceAND“stroke mortality”〉. The search terms were searched in all fields (title, abstract, keywords, and text). The search was limited to the time period 2005–2016. Around 300 articles met the initial inclusion criteria. From these around 100 articles were left after the first exclusion (based on relevance of title, abstract) and 50 after the second exclusion (based on full text and check of duplicate). After full reading of text a selection was done (based on usability of the data and comparison of study settings) and 30 articles were used as reference to this literature review.

## Abbreviations

AF: Atrial fibrillation
IS: Ischaemic stroke
OAC: Oral anticoagulation
CHA_2_DS_2_-VASc: Calculator for risk of stroke in AF patients
GP: General practitioner
NHG: Netherlands Association of General Practitioners
ESC: European Society of Cardiology
NICE: National Institute for Health and Care Excellence.
